# EELS at very high energy losses

**DOI:** 10.1093/jmicro/dfx036

**Published:** 2017-09-21

**Authors:** Ian MacLaren, Kirsty J Annand, Colin Black, Alan J Craven

**Affiliations:** School of Physics and Astronomy, University of Glasgow, Glasgow G12 8QQ, UK

**Keywords:** EELS, ELNES, EXELFS, molybdenum, zirconium, tin

## Abstract

Electron energy-loss spectroscopy (EELS) has been investigated in the range from 2 to >10 keV using an optimized optical coupling of the microscope to the spectrometer to improve the high loss performance in EELS. It is found that excellent quality data can now be acquired up until about 5 keV, suitable for both energy loss near edge structure (ELNES) studies of oxidation and local chemistry, and potentially useful for extended energy loss fine structure (EXELFS) studies of local atomic ordering. Examples studied included oxidation in Zr, Mo and Sn, and the ELNES and EXELFS of the Ti-K edge. It is also shown that good quality electron energy-loss spectroscopy can even be performed for losses above 9.2 keV, the energy loss at which the collection angle becomes ‘infinite’, and this is demonstrated using the tungsten L_3_ edge at about 10.2 keV.

## Teaser

Electron energy loss spectroscopy has been investigated in the range from 2 keV to >10 keV with 200k V electrons using an optimized microscope–spectrometer optical coupling. Excellent quality data are acquired for Zr, Mo and Sn L-edges, the Ti K-edge and the W L3 edge. Prospects for XANES/EELS correlative studies are discussed.

## Introduction

It was the vision of Mick Brown, in the hundredth year after the discovery of the electron, to build a ‘synchrotron in a microscope’ [1]. Key to his vision was that it should be possible to do many of the things hitherto only performed at synchrotrons using a modern analytical scanning transmission electron microscope. One of the key features of this idea was the understanding that electron energy-loss spectroscopy (EELS) and X-ray absorption spectroscopy (XAS) provide comparable information and probe the same key information – the unoccupied density of states in a material. Practically, however, one limitation has always been that EELS has usually been performed over a limited energy range of typically below ~2 keV. On the other hand, XAS is performed at much higher energies (typically >5 keV, as air scattering becomes significant below this, requiring adaptations like vacuum operation or low scattering gases like flowing He to allow low energy studies). Consequently, there have been very few studies with direct comparisons between XAS and EELS on exactly the same edges (Hug *et al.* [2] compared EELS and XAS for Al-K at ~1.5 keV and Vlachos *et al.* [3] did the same on the O-K edge at ~0.53 keV).

It has been possible for some while, however, to perform EELS at higher energy losses and published studies up to ~5 keV exist. For example, there are some edges in the EELS Atlas above 2 keV, such as P, Y-Ag and Os-Pb [4] as well as more recent studies of niobium L-edges [5], zirconium L-edges [6] and the titanium K-edge [7]. Unpublished data shows that Cu K edges have recently been observed using EELS with a direct electron detector in the spectrometer (P. Longo, R. Twesten, Private communication, 2017). Most of these studies have been performed using 200 keV electrons.

It may be questioned as to why such studies are relevant. After all, for example, Nb shows a strong M_4,5_ edge at 0.205 keV, as well as the L_3_ and L_2_ edges above 2.5 keV. The fact is, however, that the different edges reveal different things about the unoccupied density of states in the material. The energy loss near edge structure (ELNES) of L_3_ and L_2_ edges in a second-row transition element like Nb are dominated by dipole-allowed transitions from filled 5*p* states to empty 4*d* states – the fact that there are a lot of empty *d* states just above the Fermi level gives rise to white lines on this edge. On the other hand, the ELNES of the M_4,5_ edge is dominated by transitions from the filled 3*d* states to empty 5*p* and 5 *f* states, all of which are some way above the Fermi level giving strong lifetime broadening and a rather more rounded edge shape. So, if one is interested in the effects of oxidation, for example, this would be much more apparent in changing the density of states just above the Fermi level, and this would be much better sampled using L-edges than M-edges for this element. Similar considerations may apply for other elements, although the details of which edges are the most informative will depend on the details of the electronic structure of that element. On the other hand, for simple elemental quantification, the lowest energy edge that is well separated from other edges in that material would be ideal.

Unfortunately, acquiring high quality EELS data at higher energy losses is not simple. As Craven and Buggy [8] showed, it is possible to improve the behaviour of the post-specimen lens system in a microscope to better transfer higher energy-loss electrons into an EELS spectrometer. Some of the studies quoted above used minor tweaks of existing lens setups to improve performance in a phenomenological manner. But recent work by some of the present authors [9] has shown a method for producing vastly improved performance in transferring higher loss electrons into the spectrometer, extending the useful range for quantitative EELS out to at least 5 keV. This was done by altering the optical path of the electrons through the post-specimen lenses to make some produce virtual images as the object of the next lens, whilst other produce real images – the movement of these image positions with electron energy change is opposite and allows balancing of the effects of energy loss in the movement of the final crossover that forms the object for the spectrometer. Additionally, performance was optimized for an energy loss of 1.5 keV (not 0 keV, as for a standard imaging camera length), thus extending the range of almost constant information transfer within the spectrometer acceptance aperture to 3 keV, at the cost of a little radial distortion at higher angles (which does not affect the spectroscopy). The work reported explores what is now possible with the extended energy range for EELS investigations and offers some perspectives for the future.

## Experimental methods

All experiments presented in this manuscript were performed using a JEOL ARM200F equipped with a cold Field Emission Gun and equipped with a Gatan GIF Quantum ER EELS spectrometer equipped for fast DualEELS. Standard acquisition conditions were in STEM mode using a convergence angle of 29 mrad and a specially prepared 2 cm camera length [9] that gave a nearly constant 36 mrad acceptance angle of the 0–3 keV loss range, when used with the 2.5 mm spectrometer entrance aperture. The majority of the data was taken with a setting of the gun lens that gives approximately 600 pA of current. Some of the lower loss spectral data were recorded as spectrum images and the spectra were created from several hundred or several thousand individual spectra, after correction for energy alignment and any single channel defects (such as random X-ray spikes). Such data were generally recorded using 5 × 1 binning on the CCD in ‘high speed’ acquisition mode. This was, however, too noisy for high loss data acquisition and in such cases, a few spectra were collected and summed whilst scanning the beam over a small box of a few nm in size, each of several seconds exposure time, with 5 × 1 binning on the CCD and ‘high quality’ acquisition mode selected.

Zr and ZrO_2_ spectra were recorded from oxidized zircaloy-4 specimens, as described in more detail previously [6]. The Mo film was provided as a sputtered thin film and prepared by a standard FIB liftout method. MoO_2_ powder was purchased from Sigma Aldrich and ground in a pestle and mortar, dispersed with isopropanol and dropped onto a lacy carbon film. SnO and SnO_2_ powders were provided by AMEC Foster Wheeler (Birchwood, UK) and treated in the same way as the MoO_2_ and were the same materials as used in Hulme *et al.* [10]. Sn metal foil (98.8% purity) was purchased from Goodfellow Cambridge Ltd. (Cambridge, UK). The amorphous TiO_2_ film was produced by spin coating a Si substrate with Ti alkoxide and then annealing at 300°C, which was enough to burn out the carbon but leave the film amorphous. The W_3_Si film was co-sputtered from W and Si targets onto a Si substrate. All these latter materials were prepared for microscopy by a standard FIB liftout method.

When the energy offset required to record the edge of interest was within the 2 keV range of the drift tube voltage, the low loss and the high loss data could be acquired as spectrum images in the same dataset. While such datasets can be processed in the way described below, the edge has some defocus. Thus the low loss and the high loss were recorded sequentially as single spectra with an appropriate change of spectrum focus, *F*_*X*_, to give the sharpest edges. The energy of the high loss was aligned using the alignment of the zero-loss peak in the low loss dataset. Several sets of spectra were taken for each material and the best quality dataset used in each case, although the trends shown here have been reproduced in multiple datasets in all cases. This procedure of taking separate datasets for high and low losses at different *F*_*X*_ values also works for losses requiring an offset greater than 2 keV, since a change in the magnet current can be made to provide larger energy offsets than are possible with the drift tube alone. Backgrounds were fitted before each edge of interest and deconvolution of plural scattering was carried out using the Fourier-ratio method.

## Results and discussion

**Fig. 1.**
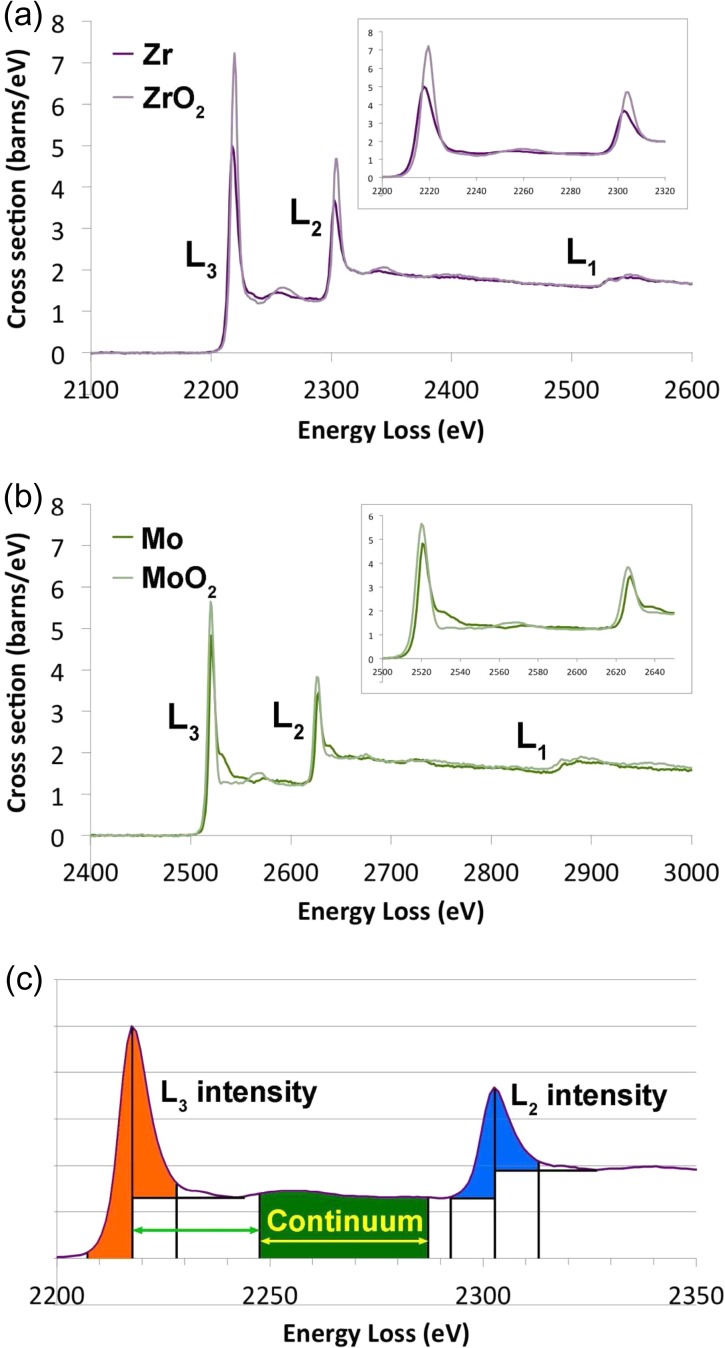
The effects of oxidation on second row transition metal L-edges: (a) Zr (*t/λ* = 0.33, 100 s acquisition) and ZrO_2_ (*t/λ* = 0.51, 100 s acquisition); (b) Mo (*t/λ* = 0.47, 40 s acquisition) and MoO_2_ (*t/λ* = 0.24, 60 s acquisition); (c) definitions of how the L_3_ and L_2_ intensities are calculated. Please note, that the vertical scale of (a) and (b) are in units of absolute differential cross section, as previously used in Craven *et al.* [12] and calculated in a similar way.

In our previous work [9], we showed that one benefit of the improved optical coupling to the spectrometer was that the continuum background was better behaved above 2 keV loss. Thus, one of the first areas that benefits from these advances is the study of oxidation in second row transition elements. Figure 1 shows the effects of oxidation on the L_3_ and L_2_ edges of zirconium and molybdenum. In both cases, the edges are displayed as background-subtracted, Fourier-ratio deconvolved edges, and are recalculated onto an absolute scale of differential cross section (in barns/eV), as in our previous work [9]. It should be noted in both Fig. 1a and b that a significant amount of the background-subtracted region before the edge is shown. This is exceptionally flat in both cases, showing that the power law extrapolation is working very well here. For three of these materials (Zr, ZrO_2_ and MoO_2_), these are the absolute numbers from a calculation of:

(1) $$\sigma =\frac{I}{I_{0}}\frac{1}{N}$$

where *N*, the areal density of atoms in the area exposed to the beam, is calculated from *nt*, where *n* is the number of atoms per unit volume (based on detailed knowledge of the crystal structure) and *t* is the sample thickness. This is obtained from the low loss using a calculated value of the mean free path for inelastic scattering, *λ* [11,12]. The Mo sample was a sputtered nanocrystalline film – if it is assumed to have the structure of bulk bcc Mo, then this would have a density of 10.22 g cm^−3^. This value, however, gave a cross section that was a little lower than expected, with the extrapolation after the L-edges about 80% of the values seen for the MoO_2_ sample. The simplest explanation was that the sputtered sample was not the full density of metallic Mo, and similar effects have previously been seen in other thin films where bulk densities are not reached. For this reason, the Mo cross section in Mo was corrected by a factor of 1.25 to allow easier comparison with that from MoO_2_. A specimen made from bulk Mo would benefit future work.

It is clear for both Zr and Mo that a small chemical shift to higher energy is noted for both the L_3_ and L_2_ edges on oxidation, as is normal for most elements. It is difficult to measure this shift accurately from this data at just 1 eV per channel. However, fitting a Gaussian on the low energy side of the L_3_ peak and defining the edge onset as the point at which the cross section reaches half the height of the white line peak, i.e. the definition of Bach *et al.* [5], the chemical shifts were found to be 1.89 eV from Zr to ZrO_2_ and 1.64 eV from MoO_2_ to Mo. (Interestingly, all these shifts were far smaller than those seen by Bach *et al.* [5] for Nb and the reason for this discrepancy is unknown.)

It should come as no surprise that the white line: continuum intensity ratio increases with oxidation in both elements, as previously observed for niobium [5]. This is to be expected due to an increase in the density of states in the 4*d* band on oxidation. It may also be noted that the Mo has a shoulder on the high-energy side of both white lines, which is lost on oxidation to MoO_2_. More subtle changes may be seen on the L_1_ edges where that for ZrO_2_ shows a peak close to the edge with a valley immediately afterwards, which is totally absent for Zr metal. More complex changes are seen on the L_1_ peak from Mo to MoO_2_.

The white line ratio changes slightly with oxidation, as previously observed for first row transition elements [13,14], and this is tabulated in Table 1. In both cases, the L_3_/L_2_ white line ratio (as defined by the background-subtracted intensity within a window of 20 eV, centred on the peak, as shown in Fig. 1c) decreases slightly on oxidation, although the trend is not strong, as previously noted for Nb by Bach *et al.* [5]. It is doubtful that such a weak trend could be used effectively for mapping oxidation state changes in materials, when the typical individual pixel data is much noisier than these long-acquisition standard spectra. The white line intensity clearly increases with oxidation for both Zr and Mo relative to the continuum background, however. This stronger trend in the white line/continuum intensity ratio could possibly be used for mapping of oxidation state.

**Table 1.** White line parameters for Zr, ZrO_2_, Mo and MoO_2_

Material	White line ratio	WL/continuum
Zr	2.17	1.009
ZrO_2_	2.09	1.286
Mo	2.19	0.891
MoO_2_	2.10	1.073

The white line ratio simply takes the ratio of the L_3_ and L_2_ white lines, each using a 20 eV window of background-subtracted intensity. The WL/continuum ratio adds the two white line intensities together and divides by the continuum intensity in a 40 eV box starting 30 eV after the L_3_ peak, in a similar manner to the previous work of Bach *et al.* [3].

**Fig. 2.**
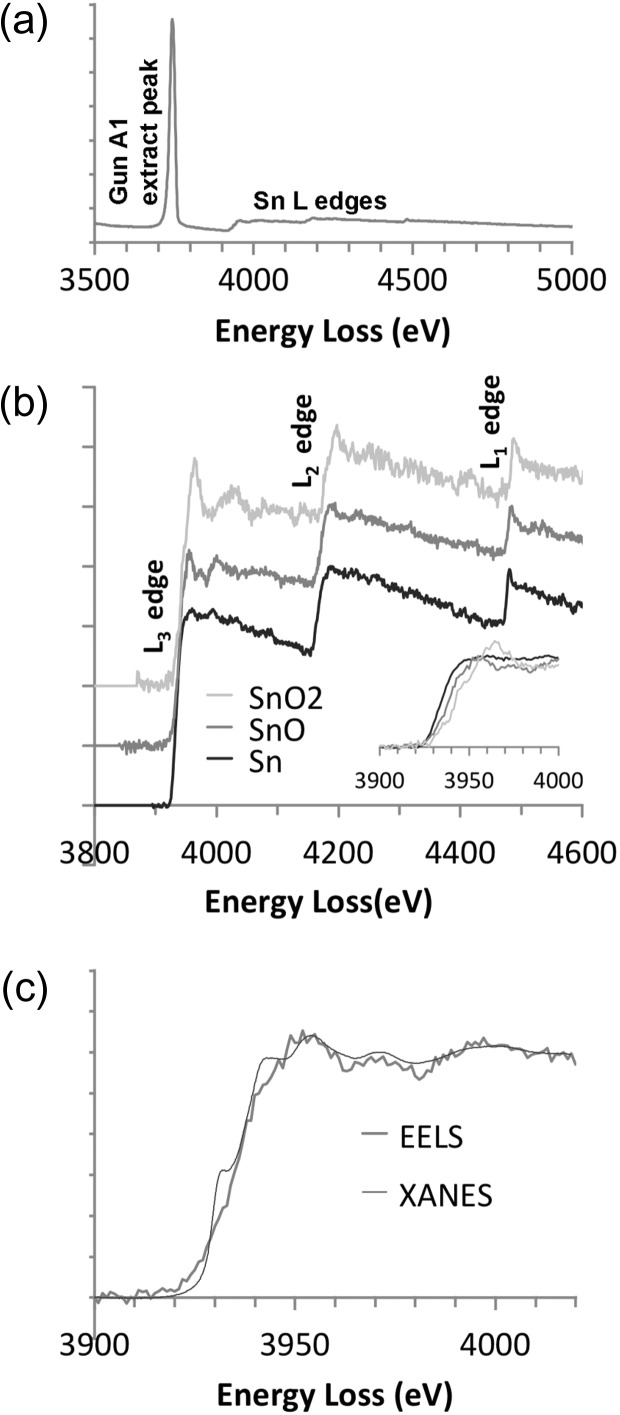
The effects of oxidation on Sn-L edges: (a) a raw spectrum for SnO; (b) background-subtracted and deconvolved edges for Sn, SnO and SnO_2_, including an inset with a detail of the chemical shifts on the L_3_ edge (*t/λ* = 0.53, 0.17 and 0.37; acquisition time 200, 150 and 150 s, respectively); (c) a comparison of EELS and XANES for the L_3_ edge of Sn in SnO, including a slight –3 eV realignment of the energy loss scale for the EELS data to match the XANES.

In making a similar comparison of the effects of oxidation on the Sn-L edges, a number of additional complications arise. The first is the peak shown in Fig. 2a. It is at ~3.720 keV, which is just in front of the Sn-L_3_ edge located at about 3.925–3.930 keV according to Hulme *et al.* [10]. This phenomenon is a feature of nearly all electron guns, e.g. McComb and Weatherly made a detailed investigation of the effect in a Schottky field emission gun where the peak intensity is much greater than in a cold field emission gun due to the higher emission current in the former [15]. The energy of the peak is directly related to the voltage on the A1 gun extraction anode, here 3.720 kV. The details of this peak and how it arises are discussed further in the Supplementary data online. At this point, the practical difficulty is that with the particular field emission tip in use, this peak lies inconveniently just before the Sn-L_3_ edge, which is located at about 3.925–3.930 keV (according to Hulme *et al.* [10]), is not terribly sharp and has significant tails. Background fitting usually made use of two fitting regions, one on either side of the peak to get a better extrapolation. However, this required some user judgement and was not entirely consistent.

Whilst deconvolution worked well, the cross section calculation was now more problematic and, whilst it generally came in within a factor of 1.5 from one sample to another, discrepancies were too high to be certain about the absolute numbers. For this reason, the spectra shown in Fig. 2 are simply normalized to have similar intensities in the continuum after the L_1_ edge to allow easy comparison. In this case, the edges show a clear trend on oxidation whereby the L_3_ and L_2_ edges have no white lines for the metal, small white lines for tin (II) oxide, and more pronounced white lines for tin (IV) oxide. This is maybe comparable to the situation in copper, which has no white lines in the metallic state but develops smaller white lines on oxidation [16,17]. In the case of tin, the 4*d* band should be totally filled for metallic tin, but if there is some hybridization of the 4*d* and 5*p* bands, it may be that there would be some vacant density of states in this band created on oxidation, resulting in the gradual appearance of the white lines. This appearance of the white lines with oxidation is also consistent with previous work by Hulme *et al.* [10] using XANES. As Hulme *et al.* [10] point out, however, there is more happening than just white line changes. A change of crystal structure on oxidation also affects these near edge structures and one other feature of interest is a broad peak after the L_3_ edge, which increases from Sn to SnO to SnO_2_. It may also be noted that there is a small peak on the L_1_ edge for all three tin samples, which changes less with oxidation. As with most other elements, slight chemical shifts to higher energy are seen for all three L edges upon oxidation, although accurate calibration is difficult at this energy, where the zero-loss peak and the L-edges cannot be recorded in the same DualEELS acquisition.

On a more detailed comparison of the current edges to the XANES work of Hulme *et al.* [10], it is seen that, although the general features of the edge are reproduced in both EELS and XANES, the XANES data is somewhat sharper at the edge. It should be noted that the spectrum focus was adjusted for the EELS data to improve the sharpness (as compared to the correct spectrum focus for the zero-loss peak). It is unclear at this point why the discrepancy exists – whether further optimization of the spectrum focus is needed, or whether other spectrometer aberrations are affecting the effective resolution at this high energy. Nevertheless, it is clear that these changes in L-edges could be used to determine oxidation states at high spatial resolution in tin in real materials, provided data can be collected for long enough to produce a sufficient signal to noise ratio. This would probably best be done by scanning small (few nm) areas whilst acquiring data to avoid beam damage and contamination on an individual point. This would still beat the spatial resolution possible with XANES, even in a microbeam setup as used, for example, by Couet *et al.* [18] where the beams were 0.2 μm × 0.2 μm. The use of counting detectors for EELS [19] would also be highly advantageous at such high energies with relatively weak signals and consequent low electron arrival rates, since all random electronic noise and readout artefacts would then be lost. This has recently been demonstrated for Cu by Longo and Twesten (Private communication, 2017).

Increasing further in energy loss, it becomes a little easier to perform good quality EELS once well above the extraction peak, since the background subtraction becomes more straightforward again. However, the problem of being unable to record the low loss and the core loss in the same DualEELS dataset persists, making the calculation of cross sections rather inaccurate. In addition, such large energy shifts involve major changes to the magnet current to provide the energy offset. If the change of energy loss with magnet current has not been carefully calibrated over such large losses (which is almost certainly true), then the absolute energy loss calibration is no longer guaranteed at higher energies. Possibly, the only solution to this is to compare the edge energies to X-ray data and perform a correction using this. Using the A1 peak is not a workable solution as the peak is too broad and is itself only known with a precision of about 10 eV from the microscope control software.

**Fig. 3.**
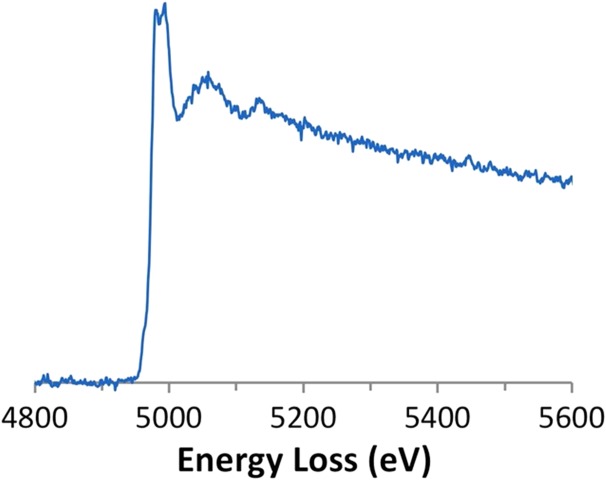
A background subtracted, deconvolved Ti-K edge from amorphous TiO_2_ (*t/λ* = 0.70, 1000 s acquisition).

Figure 3 shows the Ti-K edge from an amorphous TiO_2_ film. The background subtraction and deconvolution worked exceptionally well for this data and the quality of the edge is much better than in previous publications on Ti-K in EELS [7]. However, the edge is admittedly weak and required 1000 s of acquisition. Specifically, a strong peak is observed at the front of the edge, followed by further oscillations extending at least 200 eV after the edge. It is quite likely that if the user were prepared to count for longer, that the data quality could approach the level needed for radial distribution function analysis from the extended energy loss fine structure (EXELFS).

**Fig. 4.**
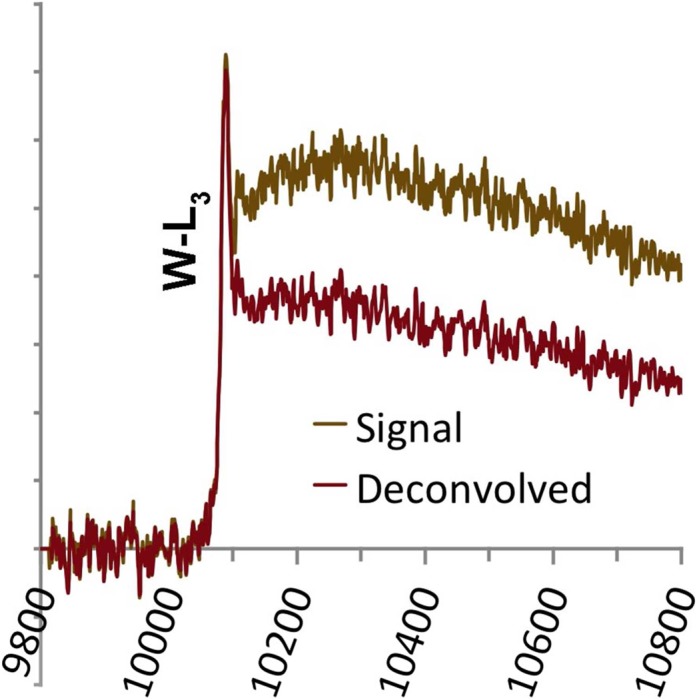
The tungsten L_3_ edge, both background-subtracted raw data and Fourier-ratio deconvolved using the low loss (*t/λ* = 0.51, 100 s acquisition).

Our previous work [9] showed that, as the energy loss increases, the position of the projector lens crossover moves away from the spectrometer with increasing energy loss eventually going off to infinity and re-appearing behind the spectrometer. For a particular energy loss, it ends up at the entrance aperture itself, resulting in an effective infinite collection angle. The actual limiting angle is restricted only by physical cutoffs in the microscope column. This energy loss is 9.2 keV in our optimized projector lens setup [9]. At this energy loss, the intensity stripe seen in the spectrometer camera view goes through a minimum width. (The energy loss at which this condition occurs can be adjusted and so there is some flexibility to allow the setup to be optimized for a specific study.). Above this energy, provided the microscope is suitably well aligned, there are still energy loss electrons for detection, although the intensity is very low. Since the crossover is now closer to the EELS entrance aperture than its position for no-loss electrons, the change in the spectrum focus, *F*_*X*_, has the opposite sign to normal. Thus, instead of increasing *F*_*X*_ to get the spectra in focus as for Figs. 1–3, *F*_*X*_ needed to be reduced to get edges above about 9.2 keV in focus. An example is shown in Fig. 4 of the W-L_3_ edge from an amorphous W_3_Si film. As before, the background shape was excellent and there is no residual shape prior to the edge after background subtraction. Deconvolution with the low loss also proceeded well without any serious problems or noise amplification. The result is an edge with a clear white line, with a narrow FWHM of 13 eV, at the onset, as expected for a third row transition element. This FWHM is slightly worse than that seen in XANES data, but certainly in the right range. The problem with the absolute energy calibration mentioned above recurs and the edge onset with our setup is found at a nominal energy 10.076 keV, when it should be 10.199 keV, according to Kaye and Laby [20]. This is likely to be mainly caused by hysteresis in the magnet but may also be affected by a non-linear component in the spectrometer dispersion at this large energy loss. It is suggested that absolute energy calibration may only be achieved by reference to X-ray data at such high energies in EELS.

**Fig 5.**
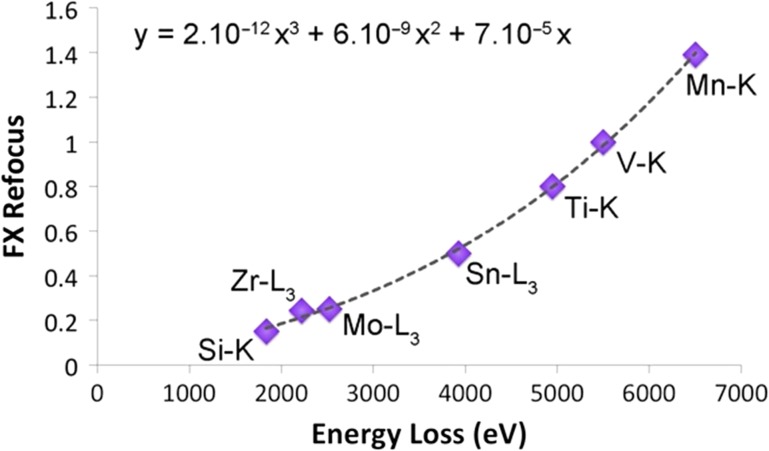
Refocus of the spectrum focus, FX, required to bring the spectrum to the sharpest possible focus as a function of energy loss.

A consistent feature of all plots shown is this work is that refocusing of the spectrum was required to achieve all these higher loss results shown in Figs. 1–4. At higher losses, since the edges were so weak that refocusing was not possible to do whilst monitoring the edge in real time, a through focal series was collected, and the best focal value was chosen for the sharpest edge. Figure 5 plots the change required from the zero-loss value of *F*_*X*_ required for best edge focus as a function of the energy loss of the edge. The behaviour was found to be fitted reasonably well by a cubic power law over this range. As the energy loss approaches the value of 9.2 keV, the energy at which the probe is focused into the centre of the EELS entrance aperture, and very close to the centre of *F*_*X*_ quadrupole, the curve will asymptotically approach infinity. To put this another way, it will be impossible to get really good quality sharp EELS data very close to the crossover energy, as the required refocus values are likely to be so high as to be unachievable in the spectrometer hardware. For this reason, the reader is advised not just to look at the spectrum but to record the camera view image for the EELS spectrum in order to understand the optical performance of the microscope-spectrometer system when operating at high-energy losses. The *F*_*X*_ value for the W-L_3_ edge is not shown on this figure. It was large and negative, since it is only just above the crossover and an *F*_*X*_ change of −7.2 gave the best results. It may be noted that, at high changes of *F*_*X*_, there may be more complex things happening in the spectrometer and that more settings are likely to require adjustment than just this one quadropole to ensure optimum performance in extreme energy EELS.

One question of interest is the optimal thickness of specimen to use for high loss EELS. Previously, Egerton [7] published Ti-K data acquired from a thick specimen (about 200 nm) of TiC, and used deconvolution to remove the plural scattering. However, signal to background is rather poor for thick specimens, and deconvolution can amplify noise unless the low loss spectrum is of exceptionally high quality. For this reason, the majority of the spectra in this work have been in a more ‘normal’ thickness range for EELS of 0.3 < *t*/*λ* < 1.0. This gives better signal to background and makes the deconvolution more noise-tolerant, even if the total signal is lower and longer acquisition times are needed for high signal to noise spectra. There is no definite rule for the best sample thickness. It is suggested that moderate thicknesses are used. To achieve acceptably low noise in both the high loss and low spectra, it is likely that the dose will have to be spread over a sufficiently large area of the specimen to limit specimen damage.

It has therefore been conclusively demonstrated that it is now possible to acquire high quality EELS data at energies more normally associated with XAS beamlines and that an era is therefore dawning in which combined correlative XAS and EELS studies could be performed on the same edges. In particular, as long as good care is taken to record a good quality low loss spectrum from the same area as the core loss data, deconvolution to a single scattering distribution works well in most cases. Stray scattering peaks from the gun structure can complicate studies of particular edges but which edges will depend on the properties of the specific emitter in use at the time and the gun mode being used. The discussion in the Supplementary data online shows that these peaks can be eliminated by suitable design of the condenser system. To perform the exact same analysis of high loss EELS as is performed in XANES would require further careful investigation of how to get the sharpest possible spectrum focus so that peak splittings and shoulders can be properly resolved. To perform very high quality radial distribution analysis from EXELFS would require higher signal to noise than was presented in this work, and it may not be possible to achieve such a high signal to noise in many materials without giving such a high radiation dose as to change the sample irreversibly. Nevertheless, with those provisos in mind, it may now be possible to consider correlative studies in the 2–10 keV range, where higher energy resolution, more accurate energy calibration, and better signal to noise is achieved in the X-ray absorption data, but higher spatial resolution, and better discrimination of different phases in an inhomogeneous sample is possible using the EELS.

**Fig. 6.**
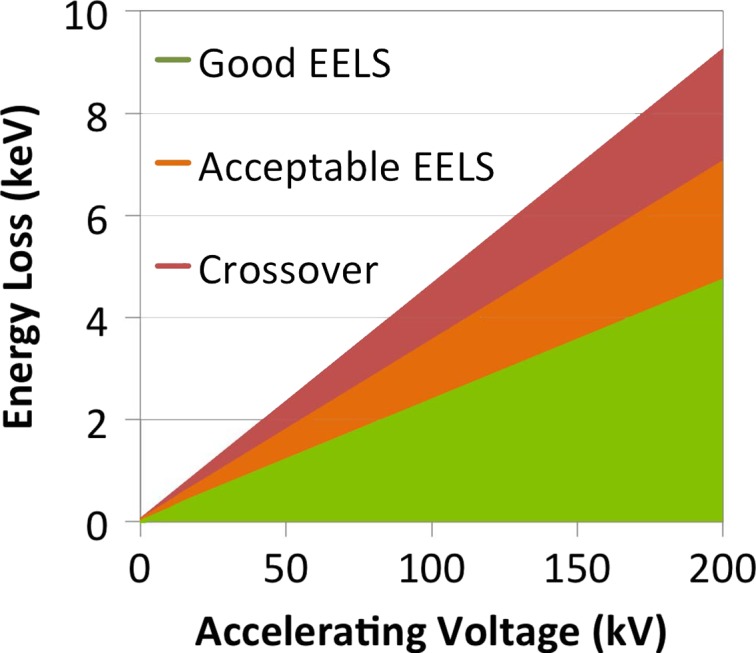
Useable ranges for EELS at different accelerating voltages extrapolated linearly from 200 kV. The ‘Good EELS’ range was defined as the range over which the acceptance angle varies by less than 5%. The Acceptable EELS range was defined as the range over which good quality data has been taken below the crossover by the authors (0–7 keV at 200 kV). The crossover was 9.2 keV at 200 kV.

This work is also of undoubted benefit to EELS in lower energy scanning transmission electron microscopy. The present work was carried out at 200 kV, but microscopes are now commonly being aligned and operated at 80, 60, 40 or even 30 kV, especially for work on 2D nanomaterials and compounds of light elements where knock-on damage thresholds are very low. All the chromatic effects seen in this work, and that have been corrected out to much higher energy than previously, will be present at much lower energies in such work. So, for instance, if it is difficult to work on the Sn-L edges at 4 keV in a microscope operated at 200 kV with *ΔE/E* = 2%, then it will be difficult to work on Si-K at 1.8 keV in a microscope operated at 80 kV and a similar optical setup. Our previous work suggests that the range of useful operation before the crossover can now be extended to a *ΔE/E* ~ 3–4% suggesting that operation out to 2 keV energy losses should now be feasible down to about 50–60 kV accelerating voltage. All this assumes a linear extrapolation from 200 kV to low voltage, which will not be quite true, after relativistic corrections have been accounted for. This is summarized in Fig. 6. Even at super-low beam energies of just 30 keV [21–23], this suggests that good quality EELS should be possible out to at least 1 keV loss.

## Conclusion

It has been shown that with the benefit of optimized coupling optics between the sample and the spectrometer, it is possible to perform high quality EELS up to above 10 keV energy loss. This can be used to study ELNES changes due to oxidation or other chemical effects, and could potentially be used for EXELFS studies of local atomic ordering, provided sufficient signal to noise ratio was present in the data. Some of the issues in performing this work are discussed, especially as regards energy calibration on the large energy shifts between the zero-loss peak and the high loss data. It is anticipated that advances in EELS detector technology will be very relevant in this area, as signal is intrinsically low and separating real signal from electronic noise then becomes critical. It is clear, however that significant progress has been made towards having a ‘synchrotron in a microscope’, and we are now entering an era in which correlative studies involving synchrotron X-ray absorption spectroscopy and high loss EELS could be more regularly performed, combining the advantages of both techniques.

## Supplementary Material

Supplementary Data
