# Supplementary material for: EELS at very high energy losses
Source: Microscopy (Oxf). 2017 Sep 21;67(Suppl 1):i78–85. doi: 10.1093/jmicro/dfx036 (PMC6025225; doi:10.1093/jmicro/dfx036)
Supplement: Supplementary Data [file highlosseelssupplementalfinalfinal.docx]

**EELS at very high energy losses**

Ian MacLaren, Kirsty J. Annand, Colin Black, Alan J. Craven

School of Physics and Astronomy, University of Glasgow, Glasgow G12 8QQ

**The gun extract peak in the spectra**

In studying the effects of oxidation on the Sn-L edges, an additional complication was present because of the gun extraction peak close to ~3.72 keV. This phenomenon is a feature of nearly all electron guns e.g. McComb and Weatherly made a detailed investigation of the effect in a Schottky gun [1]. The energy of the peak is directly related to the voltage on the A1 gun extraction anode, which was 3.72 kV for the data in the main paper.

The peak occurs when electrons from the source hit and are scattered by this electrode, which is at a lower voltage in the gun accelerator stack. Because the total current emitted by the cold field emitter used in the gun of the JEOL ARM used here is much lower than that from a Schottky field emitter, the intensities of such peaks are much lower for CFEG microscopes.

If the A1 electrode does not form the limiting aperture for the primary beam, electrons may hit one or more subsequent electrodes in the accelerator stack and peaks corresponding to their energies will also be present. Peaks from the A2 electrode in the ARM column (held at 7.52 kV in this work) are also detected.


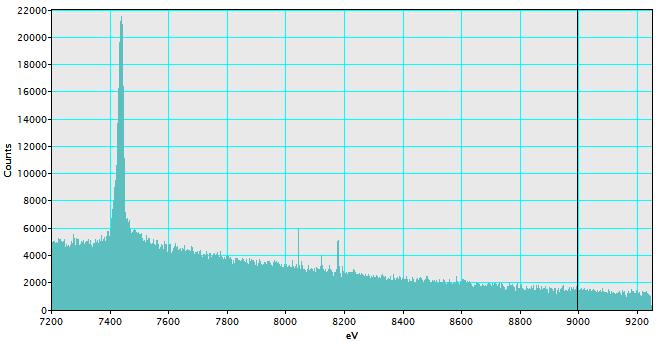


*Figure S1: An A2 extract peak from the JEOL ARM 200F.*

The explanation for the shape of the A1 peak is that the electrons scattered by the electrode have a U-shaped energy distribution. This has a maximum at close to zero, corresponding to “secondary” electrons, and one close to the original energy, corresponding to “backscattered” electrons. These electrons are then accelerated by the subsequent part of the accelerator stack. The accelerated “backscattered” electrons end up with energies similar to that of the primary electrons that pass straight through the gun without scattering. However, they represent only a tiny fraction of the number of primary electrons. Those “secondary” electrons with zero energy emerge from the accelerator stack with an energy “loss” corresponding to the energy at which they arrive at the electrode i.e. 3.72 keV in Figure 2a. Those “secondary” electrons with energies above zero will form a tail towards the zero-loss peak.


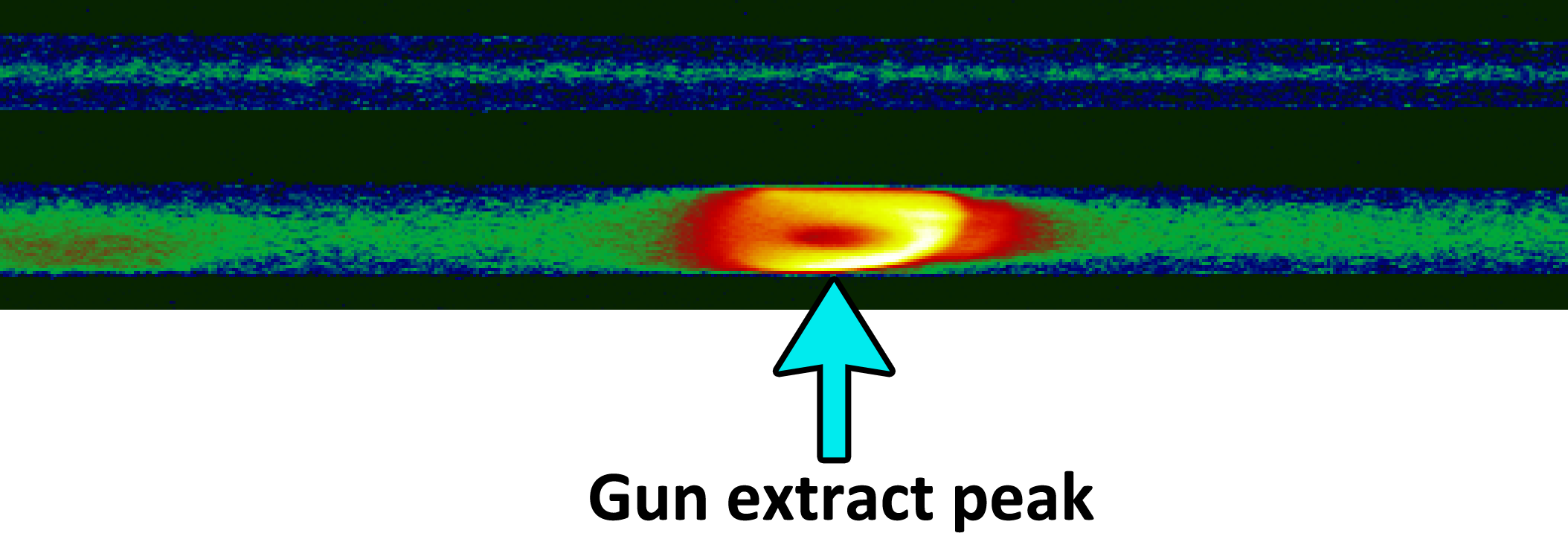


*Figure S2: The shape of the gun extract peak on the detector in the GIF Quantum (the two stripes are parts of spectra covering 3-5 keV [bottom] and 5-7 keV [top]). Note that the A1 peak is a distorted ring, not a sharp peak, for reasons discussed in the text.*

The “source” for the electrons scattered by the electrode is its edge, which is a circle significantly off the axis of the microscope, at a different axial position and at a different energy to the source of the primary electrons. When the probe is focussed on the specimen, the image of these scattered electrons will be considerably defocused and aberrated image of the ring source. Hence it will cover a very large area of specimen around the probe position. The same is true for any plane conjugate with the specimen.

The object point for the spectrometer is the projector lens cross-over, which is conjugate with the specimen in the set-up used here. Thus, the A1 peak will be broadened and not show a sharp cut-off at the energy corresponding to the A1 voltage. Additional tailing to the energy loss side will occur because some of these electrons will have undergone further inelastic events when passing through the specimen. However, this tailing will not necessarily match that of the low-loss region of the spectrum since these electrons are spread over a large area of the specimen, which may have significant thickness and composition variations with position.

Subtraction of the A1 peak with sufficient accuracy to allow good power law background subtraction over the energy range where it is present has not proved possible. Thus two background windows, preferably a wider one before the peak and a narrower one between the peak and the edge threshold are used as the fitting region for the background fit. Such a background fit gives reasonable background extrapolation. If necessary, the position of the peak can be shifted by lowering (or possibly raising) the extraction voltage. The resulting loss (or gain) of probe current can be reversed by decreasing (or increasing) the source demagnification in the condenser system albeit with some loss of spatial resolution if the demagnification is decreased.

Such peaks can be removed entirely using suitable apertures in the condenser system. One microscope, the VG Microscopes HB5, had the required apertures. In the HB5, the probe angle was defined by an aperture at the gun exit, called the virtual objective aperture. The condenser system could form a cross-over in what was termed the “selected area aperture plane”, which was prior to the scan system^[[1]](#footnote-1)^. This cross-over was then imaged onto the specimen by the objective lens. Stray scattering generated by electrons hitting the edges of the extraction electrode in the cold field emission gun and the virtual objective aperture also travelled down the column. These electrons covered a wide area around the probe and created the well known “hole count” in energy dispersive x-ray spectroscopy i.e. when the probe passed through a hole in the specimen, x-rays from the specimen and its support were still detected. These hole count x-rays were removed if a small “selected area aperture” was inserted around the probe without intersecting it [2]. It also removed the small peak in the EELS spectrum caused by scattering from the extraction electrode.

In principle, electron columns with probe correctors should be well suited to this approach since it is important that the condenser system provide a cross-over at the fixed entrance plane of the corrector. If a small aperture can be introduced in this plane and the probe aperture defined in an earlier plane, these spurious peaks would be removed and we can only hope that electron microscopy manufacturers will make modifications in due course to ameliorate such effects.

**References**

[1] McComb, D W, and Weatherly, G C (1997) The effect of secondary electrons generated in a commercial FEG-TEM on electron energy-loss spectra, *Ultramicroscopy* 68: 61-67.

[2] Craven, A J, McVitie, S, and Chapman, J N (1993) Instrumentation, techniques, and applications of electron-microscopy in the Solid-State Physics group at Glasgow-University, *Microsc. Res. Tech.* 24: 316-332.

1. Here, the “selected area aperture” is before the specimen whereas in a standard CTEM, the actual selected area aperture is after the specimen. This terminology arose in the early days of STEM because the optics and imaging of the HB5 could be understood in terms of those of the CTEM via the reciprocity theorem i.e. by interchanging the positions of the source and detector. [↑](#footnote-ref-1)
